# Metabolic Abnormalities in Normal Weight Children Are Associated with Increased Visceral Fat Accumulation, Elevated Plasma Endotoxin Levels and a Higher Monosaccharide Intake

**DOI:** 10.3390/nu11030652

**Published:** 2019-03-18

**Authors:** Anika Nier, Annette Brandt, Anja Baumann, Ina Barbara Conzelmann, Yelda Özel, Ina Bergheim

**Affiliations:** 1 Department of Nutritional Sciences, Molecular Nutritional Science, University of Vienna, A-1090 Vienna, Austria; anika.nier@univie.ac.at (A.N.); annette.brandt@univie.ac.at (A.B.); anja.baumann@univie.ac.at (A.B.); 2 Department of Nutritional Medicine, (180), University of Hohenheim, D-70599 Stuttgart, Germany; ina.conzelmann@gmx.de (I.B.C.); yelda@directbox.com (Y.Ö.)

**Keywords:** fructose, intestinal permeability, children, normal weight, dietary pattern

## Abstract

Being overweight has been identified as the main risk factor for the development of metabolic disorders in adults and children. However, recent studies suggest that normal weight individuals are also frequently affected by metabolic abnormalities with underlying mechanisms not yet fully understood. The aim of the present study was to determine if dietary pattern and markers of intestinal permeability, as well as inflammation, differ between normal weight healthy children and normal weight children suffering from metabolic abnormalities. In total, 45 normal weight children aged 5–9 years were included in the study, of whom nine suffered from metabolic abnormalities. Anthropometric data, dietary intake and markers of inflammation, as well as intestinal permeability, were assessed in fasting blood samples. Neither BMI nor BMI-SDS differed between groups; however, children with metabolic abnormalities had a significantly larger waist circumference (+~5 cm) and a higher leptin to adiponectin ratio. While plasma leptin levels are significantly higher in normal weight children with metabolic abnormalities, neither TNF α nor sCD14, adiponectin, PAI-1 or IL-6 plasma levels differed between groups. Despite similar total calorie and macronutrient intake between groups, mean total fructose and total glucose intake (resulting mainly from sugar sweetened beverages, fruits and sweets) were higher in children with metabolic abnormalities than in healthy children. Time spent physically active was significantly higher in healthy normal weight children whereas time spent physically inactive was similar between groups. Furthermore, bacterial endotoxin levels were significantly higher in the peripheral plasma of normal weight children with metabolic abnormalities than in healthy normal weight children. Our results suggest that metabolic disorders in normal weight children are associated with a high monosaccharide intake and elevated bacterial endotoxin as well as leptin plasma levels, the latter also discussed as being indicative of visceral adiposity.

## 1. Introduction

In adults as well as children and adolescents (for overview see [1]), overweightness and obesity have been identified as key risk factors for the development of metabolic abnormalities like hypertension, insulin resistance and dyslipidemia, but also for non-alcoholic fatty liver disease (NAFLD). Indeed, recent studies suggest that ~70% of overweight adults suffer from one or more metabolic abnormalities, with body weight showing a strong correlation with the prevalence and number of metabolic abnormalities [2]. Similar numbers and associations have also been reported for children and adolescents [3,4]. However, studies also suggest that metabolic abnormalities are not only found in overweight or obese children, but that ~20% of normal weight children, depending on reference values and definition used, display one or more signs of metabolic impairments [5,6]. Metabolic diseases in the latter group of children often remain undetected, as normal weight is still frequently thought to be associated with optimal metabolic health, thereby increasing the probability of these children developing more severe stages of disease before it being detected. However, pediatric metabolic disorders are suggested to increase the risk for metabolic diseases, atherosclerosis and even type 2 diabetes later in life by 2–3 fold [7,8]. Furthermore, it has been suggested that insulin sensitivity decreases over time in normal weight children with one or more metabolic risk factors while being maintained in healthy normal weight children [9]. The mechanisms involved are not yet fully understood.

More than 30 years ago, Ruderman et al. described “metabolically-obese” normal-weight adult individuals [10,11,12], referring to individuals with a normal body weight and BMI < 25 kg/m^2^ but an increased body fat >30% (for overview also see [13,14]) and a markedly higher prevalence of metabolic abnormalities than normal weight individuals with body fat <30%. In recent years, it has been suggested that a similar phenotype is also found among children and adolescents (for overview also see [15]) and that this is also associated with a higher likelihood to suffer from metabolic abnormalities [16]. The results from Yaghootkar et al. suggest that there is a genetic link between certain metabolic diseases, including insulin resistance, hypertension, coronary artery disease and type 2 diabetes, with a higher visceral-to-subcutaneous adipose tissue ratio in normal weight individuals [17]; still, genetic predisposition is not sufficient to explain all of the metabolic abnormalities found in normal weight children. Indeed, results in recent years also suggest that alterations of intestinal microbiota and barrier function, as well as dietary pattern, could be critical (for overview see [18]). For instance, results of Alisi et al. [19], Dasu et al. [20] and our own group [21,22,23] have shown that overweight adult and juvenile patients with type 2 diabetes and non-alcoholic fatty liver disease (even at very early stages of the disease) have altered intestinal barrier function and elevated bacterial endotoxin levels. Furthermore, a diet rich in saturated fatty acids and mono- and disaccharides has repeatedly been proposed to be critical in the development of metabolic diseases in overweight adults but also children [23,24,25,26,27].

Starting from this background, the aim of the present study is to determine if dietary pattern, markers of intestinal permeability and inflammation differ between normal weight metabolically unhealthy (MUH) children and normal weight metabolically healthy (MH) children.

## 2. Materials and Methods 

### 2.1. Subjects

A total of 45 normal weight children aged 5–9 years were recruited from the so-called “Hohenheim Fructose Intervention (HoFI) study” registered at http://clinicaltrials.gov (NCT01306396) and were analyzed in the present study. The prevalence of metabolic disorders was assessed using a score as previously described by Maier et al. [4]. This score was modified from Weiss et al. [28] and composed of BMI >97th percentile, triglyceride levels >95th percentile, HDL-cholesterol level <5th percentile, systolic and/or diastolic blood pressure >95th percentile and impaired glucose tolerance as well as the prevalence of hepatic steatosis, as determined by ultrasound.

The ethics committee of the Landesärztekammer Baden-Württemberg, Stuttgart, Germany, approved the present study, which was then performed in accordance with the ethical standards laid down in the Declaration of Helsinki (2008). Before being enrolled in the study, all subjects and their guardians gave written informed consent to participate in the study. 

Inclusion and exclusion criteria have previously been summarized by Engstler et al. [29] and Maier et al. [4]. Briefly, children included in our study had no known history of (i) steatohepatitis; (ii) renal insufficiency; (iii) diabetes type 1 and 2; (iv) chronic diseases of the gastrointestinal tract; or (v) taking lipid-lowering drugs or drugs affecting lipid metabolism at the time of recruitment.

### 2.2. Assessment of Dietary Intake, Leisure Time Physical Activities and Anthropometric Measurements

Dietary intake, anthropometry and sportive and sitting leisure time activities were assessed as previously described [30]. In brief, dietary intake of children was assessed in the presence of their respective guardians by an experienced nutritionist through two 24 h dietary recalls, which were carried out on two independent days including one weekday and one weekend day. Using these data, nutritional intake and dietary pattern were determined using the software EBISpro (Version 8.0, 2007, Germany). Physical and sedentary activities in spare time were assessed using a questionnaire (adapted and modified from KiGGS [31]). To determine age and gender specific BMI percentiles as well as BMI standard deviation scores (BMI-SDS), reference data for German children to categorize normal weight and overweight in children were used [32]. 

### 2.3. Blood Pressure, Abdominal Ultrasound and Glucose Metabolism

Systolic and diastolic blood pressure as well as liver status using ultrasound were assessed and categorized as described previously [4]. In accordance with the reference values of the German Diabetes Association (also see: www.deutsche-diabetes-gesellschaft.de), impaired glucose tolerance was defined as glucose concentrations >140 mg/dL at 120 min after oral glucose ingestion. The homeostasis model assessment for insulin resistance (HOMA-IR) index (formula: HOMA-IR = [fasting insulin (µIU/mL) × fasting glucose (mmol/L)]/22.5) was used to determine insulin resistance.

### 2.4. Laboratory Measurements 

A fasting venous blood sample was taken to analyze activity of alanine aminotransferase (ALT), aspartate aminotransferase (AST) and blood glucose in a routine laboratory (Sindelfingen, Germany). Concentrations of active plasminogen activator inhibitor (PAI)-1 (LOXO, Dossenheim, Germany), leptin, insulin and soluble CD14 (sCD14) (all Hölzel GmbH, Wildberg, Germany), c-reactive protein (CRP) (DRG Instruments GmbH, Marburg, Germany), interleukin 6 (IL-6) (R&D Systems, Abingdon, UK), lipopolysaccharide-binding protein (LBP) (Abnova, Taipei City, Taiwan), and tumor necrosis factor alpha (TNF α) (IBL international GmbH, Hamburg, Germany) were determined in plasma and serum, respectively, using commercially available ELISA kits. 

### 2.5. Endotoxin Concentrations and Small Intestinal Bacterial Overgrowth

To assess endotoxin concentrations, plasma samples were heated at 70 °C for 20 min and an endpoint enzymatic assay based on limulus amebocyte lysate (Charles River, Ecully, France) was performed as detailed previously [21]. Recovery rates of the LAL test were 112% on average in the present study. An H_2_ breath test was performed to determine small intestinal bacterial overgrowth (SIBO) as described by Volynets et al. [23]. 

### 2.6. Statistical Analysis

Outliers determined by Grubbs test were excluded from further analysis. Student’s *t*-tests and Mann-Whitney *U* tests were used to determine statistical significant differences between groups. Differences in sex, ethnicity and SIBO were assessed using Fisher’s exact test (GraphPad Prism, version 7.03, 2017, GraphPad Software Inc., San Diego, CA, USA). A *p*-value ≤ 0.05 was defined as statistically significant. Data are shown as total numbers or mean ± standard error of the mean (SEM). 

## 3. Results

### 3.1. Anthropometry and Metabolic Characteristics of Participants

**Figure 1:**
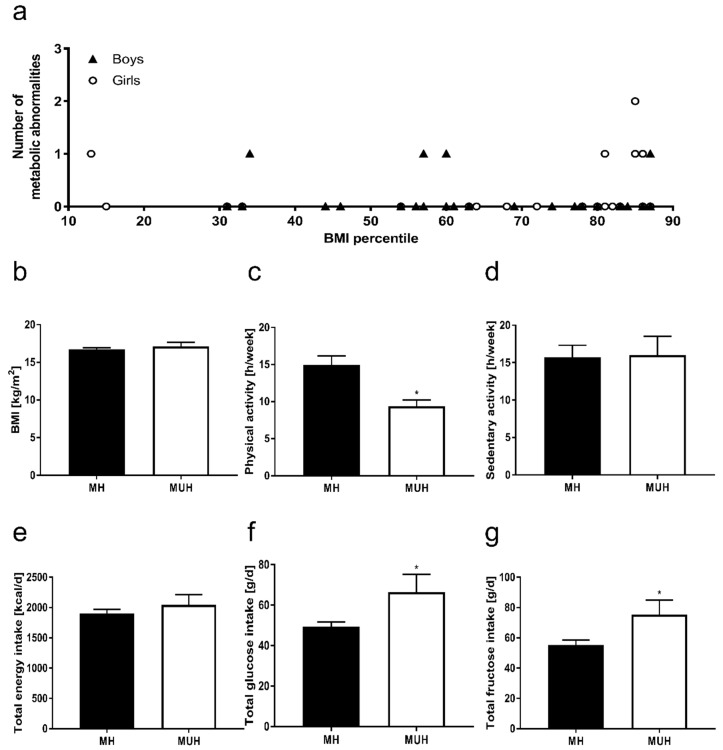
(**a**) Number of metabolic abnormalities in boys and girls of both groups, (**b**) BMI, (**c**) physical and (**d**) sedentary activities, (**e**) total energy, (**f**) total glucose and (**g**) total fructose intake in metabolically healthy children (MH) and children suffering from metabolic abnormalities (MUH). Total glucose and total fructose derived from sucrose and free glucose and fructose, respectively. Data are shown as mean ± SEM, * *p* ≤ 0.05 compared to healthy children.

No differences were found between groups regarding pubertal status, ethnicity and parental BMI. As shown in Figure 1, eight of the 45 normal weight children enrolled in the study showed at least one metabolic abnormality, while one child was even found to suffer from two metabolic abnormalities (20% of children enrolled). Of the nine children with metabolic abnormalities, four suffered from elevated blood pressure, two had elevated triglyceride serum concentrations, one child showed HDL serum concentrations <5th percentile, and three children showed early signs of NAFLD (e.g., steatosis grade 1 as determined by ultrasound). None of the nine children displayed any signs of impaired glucose tolerance as assessed by an oral glucose tolerance test. Still, as both insulin serum levels and HOMA-IR were significantly higher in MUH children when compared to MH children (+~33% and +~37%, respectively; *p* < 0.05 for both), it can be assumed that at least some of the MUH children suffered from early stages of insulin resistance. MH and MUH children neither differed in body weight, height, BMI nor BMI-SDS (see Table 1, Figure 1). However, mean waist circumferences of MUH children were higher (+~5 cm) than those of MH children. No differences between groups were found when comparing waist-to-height ratios (see Table 1). 

Both mean systolic and diastolic blood pressure were significantly higher in MUH children than in MH children (+~7 mmHg and +~8 mmHg, respectively; *p* < 0.05 for both). Triglyceride concentration in serum was significantly higher in MUH children when compared to healthy ones (+35%, *p* = 0.05). While HDL cholesterol levels were similar between groups, total cholesterol and LDL cholesterol concentrations in serum were by trend higher in MUH children than in those without metabolic abnormalities (total cholesterol, +~13%; LDL cholesterol, +~10%; *p* = 0.07 for both). No differences between groups were found when comparing liver transaminase activities in serum (see Table 1).

### 3.2. Pro-Inflammatory Markers and Adipokines in Blood of Normal Weight Children with and without Metabolic Abnormalities

**Figure 2:**
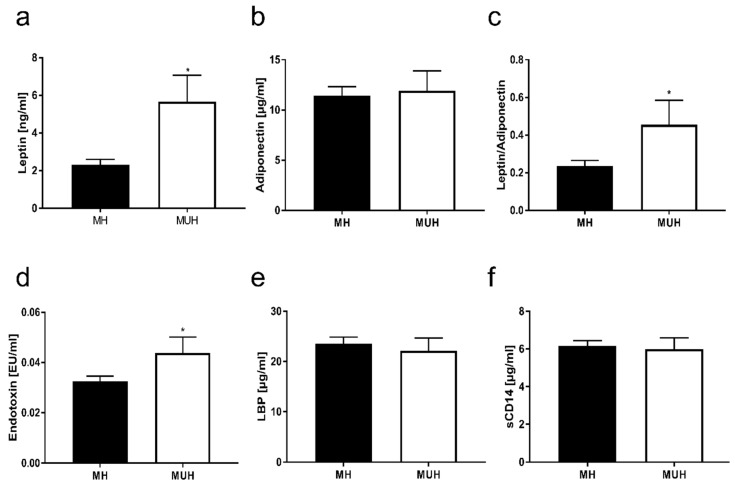
(**a**) Leptin and (**b**) adiponectin plasma levels, (**c**) leptin/adiponectin ratio, (**d**) endotoxin plasma levels, (**e**) LBP plasma and (**f**) sCD14 serum levels in metabolically healthy children (MH) and children suffering from metabolic abnormalities (MUH). Data are shown as mean ± SEM, * *p* ≤ 0.05 compared to healthy children. LBP: Lipopolysaccharide binding protein, sCD14: soluble CD14.

TNFα, IL-6, active PAI-1, adiponectin and CRP protein levels in plasma were similar between groups (see Table 2, Figure 2). In contrast, plasma leptin concentrations were significantly higher in MUH children than in MH children (+2.3-fold, see Figure 2). In line with these findings, leptin to adiponectin ratio, having been suggested to be indicative of visceral fat accumulation [33], was also significantly higher in MUH children than in those without metabolic disorders (+2.6-fold, see Figure 2).

### 3.3. Markers of Intestinal Permeability in Blood of Normal Weight Children with and without Metabolic Abnormalities

The prevalence of SIBO was similar between normal weight children with and without metabolic abnormalities (see Table 1). In contrast, bacterial endotoxin levels were significantly higher in plasma of MUH children than in MH ones. LBP plasma levels and concentrations of soluble CD14 were similar between groups (see Figure 2).

### 3.4. Nutritional Intake and Physical Activity of Normal Weight Children with and without Metabolic Abnormalities

Neither total energy, fat and protein intake nor total carbohydrate and fiber consumption differed between MH and MUH children (see Table 3, Figure 1). However, intake of carbohydrate varied considerably within the group of MUH children. Average intake of polyunsaturated fatty acids, monounsaturated fatty acids and saturated fatty acids, as well as of animal and plant derived protein, was similar between groups. While mean intake of starch was also similar between groups, MUH children consumed significantly more total glucose (+~17 g/day) and total fructose (+~20 g/day) per day when compared to MH children (Table 2, Figure 1). Both, glucose and fructose were derived from sucrose and free glucose and fructose, respectively. Mean spare time spent with sedentary activities such as reading, doing handicrafts, playing computer games and watching TV was similar between groups, whereas mean spare time spent physically active was significantly greater in MH children than in MUH children (+~3 h/week, *p* < 0.05, see Figure 1).

To further delineate the source of the elevated monosaccharide intake of MUH children, dietary patterns of children were analyzed. The percentage of children reporting to consume beverages, fruits and vegetables, bread and bakery goods, potatoes, pasta and rice, milk and milk products, meat, oils, butter, spreads and sweets was similar between groups (data not shown). However, when comparing energy intake derived from these foods in children reporting to consume these foods, MUH children were found to have a significantly higher intake of beverages and fruits/dried fruits, as well as sweets and sugar (*p* ≤ 0.05 for all parameters), compared to MH children (see Table 4).

## 4. Discussion

While results obtained in more recent years in studies with normal weight adults with one or more metabolic abnormalities (e.g., elevated blood triglycerides and systolic blood pressure) suggest that these individuals have higher odds of developing type 2 diabetes or severe cardiovascular complications than healthy normal weight individuals [34], knowledge of the mechanisms involved in the development of normal-weight associated metabolic abnormalities, especially in children, is rather limited. Despite a similar BMI and BMI-SDS, MUH children had a higher waist circumference, higher leptin levels and higher leptin to adiponectin ratios, all being indicative of a visceral fat accumulation when compared to MH children [33]. These findings are in line with those of others in adults [34,35], further suggesting that metabolic abnormalities in normal-weight individuals may indeed not only be associated with an overall greater fat mass, but also a visceral fat accumulation. Furthermore, while fasting glucose and glucose tolerance was not altered, fasting plasma insulin levels and HOMA-IR of MUH children were both significantly higher than in MH children. Studies in rodents and humans suggest that impairments of glucose tolerance may develop secondarily to visceral and ectopic fat deposits [36,37,38]. In support, results of an animal study by Bursac et al. [39] suggest that among the adverse metabolic effects described for an elevated fructose intake like insulin resistance and the metabolic syndrome [40], an increased accumulation of visceral adipose tissue is a key factor. However, it cannot be ruled out that some of the children already suffered from early stages of insulin resistance, as mean fasting insulin levels and HOMA-IR were both already above those of children without metabolic abnormalities. Katsuki et al. [41] previously reported that in metabolically obese, normal weight adult Japanese individuals, the degree of visceral fat is positively associated with insulin resistance as assessed by euglycemic-hyperinsulinemic clamp studies, while in the same study glucose tolerance test revealed no differences between groups. Indeed, a glucose tolerance test has been suggested before to be less sensitive than the euglycemic clamp technique [42].

Interestingly, TNF α, IL-6, PAI-1 and CRP serum levels, which have all been suggested in previous studies to be associated with the development of metabolic abnormalities in overweight adults and children [25,43,44], were similar between groups. These data somewhat contrast with the findings of others [45], which showed that in normal weight children with metabolic abnormalities, pro-inflammatory markers are elevated when compared to healthy children. Differences from this study and ours might have resulted from differences in population (Spanish vs. German children) and using different definitions for metabolic health status (Olza et al. [46] vs. Weiss et al. [28] in the present study). Moreover, in the present study children only showed early signs of metabolic alterations, so it cannot be ruled out that over time inflammatory markers will increase. 

### 4.1. Metabolic Abnormalities in Normal Weight Children Are Associated with a Monosaccharide-Rich Diet and Lower Physical Activity

Various environmental, social and behavioral factors are discussed as possible risk factors for the development of metabolic abnormalities in overweight children [47]. In recent years, a diet rich in carbohydrates, herein particularly a diet rich in fructose or sugar sweetened beverages [25,48], but also low physical activity [49,50,51], have been suggested to be associated with the increased prevalence of overweight and the development of different metabolic disorders including insulin resistance, NAFLD and dyslipidemia in children and adolescents. In the present study, MUH children were found to consume more sugar sweetened beverages, fruits, dried fruits, sweets and added sugars, resulting in a significantly higher intake of both total glucose and total fructose, compared to MH children. Total carbohydrate intake was similar between groups. Besides differences in dietary pattern, the small sample size might have resulted in the lack of differences in total carbohydrate intake between the two groups. Also, physical activity was significantly higher in MH children than in MUH children, further suggesting that similar to the findings in overweight children, dietary monosaccharide intake and a lack of physical activity may be critical in the development of metabolic abnormalities [25,30]. Studies in humans and animal models suggest that visceral fat accumulation may increase depending on the kind of sugar consumed [52,53]. For instance, Stanhope et al. [53] found that in overweight and obese adults, chronic intake of high amounts of fructose is associated with increases in visceral adipose fat mass and beginning insulin resistance, while in the same study similar effects were not found when isocaloric amounts of glucose were consumed. However, whether an elevated intake of fructose and glucose in children over an extended period of time, as was probably found in the children enrolled in the present study, is associated with an increased visceral adipose tissue mass has, to our knowledge, not yet been studied systematically. Taken together, our results suggest that both dietary monosaccharide intake and physical activity may be critical in the development of metabolic abnormalities and visceral fat accumulation in normal weight children. 

### 4.2. Metabolic Abnormalities in Normal Weight Children Are Associated with Altered Intestinal Barrier Function

Results of animal studies and some human studies suggest that an increased intake of sugar and especially fructose is associated with impairments of intestinal barrier function and, subsequently, results in an increased translocation of bacterial endotoxin [25,54,55]. Indeed, it was recently shown by us that in overweight children with early signs of NAFLD both fructose intake and bacterial endotoxin plasma levels are significantly higher than in overweight children without NAFLD [25]. In the present study, bacterial endotoxin concentrations were also significantly higher in MUH children than in MH children. Results of animal studies suggest that the elevated bacterial endotoxin levels found after intake of a fructose-rich diet are related to a loss of tight junction proteins in the upper parts of the small intestine [54,56]. Treatment with antibiotics, at least in animal models, has been suggested to prevent the onset and progression of metabolic diseases in model organisms [52,54,57]. However, whether a loss of tight junction proteins and changes of intestinal barrier function in the small intestine shown in animal models after chronic fructose intake is also involved in the elevated bacterial endotoxin levels found in the present study remains to be determined. Indeed, it has been suggested that clearance of bacterial endotoxin due to an impaired liver function may contribute to elevated bacterial endotoxin levels in patients with severe liver damage (for overview see [58]). However, in the present study none of the children showed any signs of severe liver disease. In contrast to the findings for endotoxin levels and previous findings in overweight children with and without metabolic abnormalities [25], LBP plasma levels did not differ between groups in the present study. Differences between previous findings and the ones in the present study might have resulted from the children’s weight status (overweight vs. normal weight children in the present study). Indeed, it has been shown before that circulating LBP levels are associated with obesity-related insulin resistance [59]. Moreover, in the previous study all children suffered from non-alcoholic fatty liver disease, where LBP has been suggested to be a critical development factor [60], while in the present study only three children showed early signs of NAFLD. Taken together, results of the present study further underline that elevated bacterial endotoxin levels may be critical in the development of metabolic diseases. However, our results by no means preclude that other factors including a genetic predisposition may also be critical in the onset of metabolic abnormalities in normal weight children.

### 4.3. Limitations

Our study is not without its limitations, which need to be considered when interpreting the results. The sample size was rather small and consisted mainly of Caucasian children. Therefore, it cannot be ruled out that findings might differ in larger populations with different ethnic backgrounds. Still, the findings of our study are in line with findings of others and previous findings of our own group in adults and children, showing that metabolic impairments are associated with a higher fructose intake, visceral adiposity and alterations in intestinal barrier function [25,40,41,53]. Furthermore, as nutritional intake and physical activity during leisure time were self-reported, misreporting or recording errors might have occurred, as discussed previously by others [61,62]. Physical activity was assessed using questionnaires only assessing spare time activities (e.g., physical and sedentary activities), while activities included in the daily routine like time spent at school, eating or sleeping, were not assessed. Therefore, it cannot be ruled out that some activities, be they sedentary or physical, were missed. However, in a pilot study performed before (unpublished data), 24 h recalls and questionnaires to assess physical and sedentary activities were found to be the most suitable tool to assess dietary intake and physical activities in children below the age of 10. For ethical and compliance reasons it was not possible to assess visceral adipose tissue with imaging methods. It cannot be ruled out that some of the differences found in waist circumference might have been attributed to the slightly higher age of MUH children (+0.7 year, n.s.). Indeed, it has been shown before in children that waist circumference increases with age [63]; however, between the age of 5 and 9 years this increase amounts for ~1–2 cm of the increase of waist circumference per year, in both girls and boys. As the study depended highly on the compliance of children enrolled, as well as their guardians, intestinal barrier dysfunction was only assessed by indirect markers like plasma endotoxin levels and a glucose breath test for H_2_ exhalation to determine SIBO, rather than by more direct markers like Xylose excretion or PEG requiring an additional fasting period and urine collection over an extended period of time. Another limitation of the study is that for ethical reasons, and as the study was carried out in a non-clinical setting, we were not able to carry out repeated blood collections and an euglycemic-hyperinsulinemic clamp to determine insulin resistance in children, the latter being considered the gold standard for determination of insulin resistance [42,64,65]. Nevertheless, previous studies have shown that HOMA-IR may reflect insulin sensitivity in a strong association with clamp-measured total glucose disposal [66]. 

## 5. Conclusions

Taken together, results of the present study suggest that in normal weight children metabolic abnormalities (e.g., hypertension, hypercholesterinemia, and early signs of NAFLD) are associated with a higher waist circumference and leptin to adiponectin ratio, both being indicative of an increased visceral fat mass [33]. Indeed, visceral fat mass is discussed to be associated with the development of metabolic diseases in children [67]. However, our data also suggest that despite similar total energy intake, dietary pattern and nutritional intake and herein especially the intake of fructose and glucose differs markedly between normal weight children with and without metabolic abnormalities. Furthermore, our data also suggest that these differences in nutritional intake are associated with increased markers of intestinal permeability. Whether or not targeting nutritional habits, for instance through dietary interventions focusing on reducing monosaccharide intake or intestinal barrier function through the intake of pre- and pro-biotics, has beneficial effects on the prevalence of metabolic abnormalities in normal weight children as well as molecular mechanisms involved needs to be determined in future studies. 

## Figures and Tables

**Table 1:** Characteristics of healthy children and children suffering from metabolic disorders.

	MH	MUH
*n*	36	9
Sex (male/female)	20/16	4/5
Ethnicity (Caucasian/Asian)	27/9	6/3
Age (years)	7.3 ± 0.2	8.0 ± 0.4
Weight (kg)	27 ± 1	29 ± 5
Height (m)	1.26 ± 0.01	1.30 ± 0.02
BMI (kg/m^2^)	16.7 ± 0.2	17.1 ± 0.5
BMI-SD score	0.4 ± 0.1	0.4 ± 0.3
Waist circumference (cm)	59 ± 1	64 ± 2 *
Waist to Height Ratio	0.48 ± 0.01	0.48 ± 0.01
Systolic blood pressure (mmHg)	103 ± 1	109 ± 3 *
Diastolic blood pressure (mmHg)	62 ± 1	70 ± 3 *
ALT (U/L)	19 ± 1	20 ± 3
AST (U/L)	33 ± 1	33 ± 2
Triglycerides (mg/dL)	57 ± 3	77 ± 13 *
HDL cholesterol (mg/dL)	57 ± 1	56 ± 3
LDL cholesterol (mg/dL)	100 ± 3	113 ± 4
Total cholesterol (mg/dL)	170 ± 4	187 ± 3
Insulin (µIU/mL)	9 ± 0.4	12 ± 1 *
Fasting Glucose	85 ± 0.9	87 ± 2.3
HOMA-IR	1.9 ± 0.1	2.6 ± 1.8 *
SIBO (with/without) ^#^	2/32	0/7

Data are shown as absolute numbers or mean ± SEM, * *p* ≤ 0.05 compared to healthy children, ^#^ two children of each group refused the H_2_ exhalation test. BMI: body mass index, BMI-SD score: BMI standard deviation score, ALT: alanine aminotransferase, AST: aspartate aminotransferase, HOMA-IR: homeostatic model assessment for insulin resistance, MH: metabolically healthy children, MUH: metabolically unhealthy children, SIBO: small intestinal bacterial overgrowth.

**Table 2:** Proinflammatory Markers and CRP of healthy children and children suffering from metabolic disorders.

	MH	MUH
TNF α (pg/mL)	0.12 ± 0.004	0.12 ± 0.007
IL 6 (pg/mL)	0.68 ± 0.12	1.12 ± 0.47
PAI-1 (U/L)	6.8 ± 0.8	8.7 ± 1.0
CRP (mg/L)	0.36 ± 0.12	0.39 ± 0.17

Data are shown as mean ± SEM. TNF α: Tumor necrosis factor alpha, IL 6: Interleukin 6, MH: metabolically healthy children, MUH: metabolically unhealthy children, PAI-1: Plasminogen Activator Inhibitor-1, CRP: c-reactive protein.

**Table 3:** Nutritional Intake of healthy children and children suffering from metabolic disorders.

	MH	MUH
Total Energy Intake (kcal/day)	1900 ± 70	2043 ± 168
Total Fat Intake (g/day)	78 ± 4	72 ± 8
PUFA (g/day)	10 ± 1	8 ± 1
MUFA (g/day)	21 ± 1	18 ± 3
SFA (g/day)	29 ± 2	22 ± 3
Total Protein Intake (g/day)	59 ± 3	60 ± 6
Animal Protein (g/day)	27 ± 2	30 ± 4
Plant Protein (g/day)	16 ± 1	20 ± 2
Total Carbohydrate Intake (g/day)	242 ± 10	280 ± 30
Starch (g/day)	99 ± 6	96 ± 11
Fructose (g/day) ^§^	55 ± 3	76 ± 10 *
Glucose (g/day) ^§^	49 ± 2	67 ± 9 *
Fiber Intake (g/day)	15 ± 1	17 ± 2

Data are shown as mean ± SEM, * *p* ≤ 0.05 compared to healthy children. MH: metabolically healthy children, MUH: metabolically unhealthy children, PUFA: polyunsaturated fatty acids, MUFA: monounsaturated fatty acids, SFA: saturated fatty acids. ^§^ deriving from sucrose and free glucose and fructose, respectively.

**Table 4:** Dietary patterns of healthy children and children suffering from metabolic disorders.

	MH	MUH
Beverages (kcal/day)	161 ± 18	265 ± 47 *
Fruits/Dried Fruits (kcal/day)	86 ± 12	148 ± 26 *
Vegetables/Legumes (kcal/day)	27 ± 5	22 ± 4
Potatoes/Pasta/Rice (kcal/day)	210 ± 33	194 ± 46
Bread (kcal/day)	259 ± 22	239 ± 50
Spreads (kcal/day)	141 ± 23	79 ± 27
Bakery Goods (kcal/day)	206 ± 28	210 ± 54
Meat (kcal/day)	216 ± 24	156 ± 19
Milk and Dairy (kcal/day)	161 ± 16	204 ± 54
Cheese and Quark (kcal/day)	92 ± 15	39 ± 17
Oils, Margarines and Butter (kcal/day)	94 ± 12	65 ±12
Sweets and Sugar (kcal/day)	154 ± 26	239 ± 38 *

Data are shown as mean ± SEM, * *p* ≤ 0.05 compared to healthy children. MH: metabolically healthy children, MUH: metabolically unhealthy children.
